# Modelo para la inspección, vigilancia y control sanitario con enfoque de riesgos en Colombia

**DOI:** 10.26633/RPSP.2017.105

**Published:** 2017-07-20

**Authors:** Álvaro Aroca, Javier Guzmán

**Affiliations:** 1 Instituto Nacional de Vigilancia de Medicamentos y Alimentos Instituto Nacional de Vigilancia de Medicamentos y Alimentos Bogotá Colombia Instituto Nacional de Vigilancia de Medicamentos y Alimentos, Bogotá, Colombia.

**Keywords:** Riesgo sanitario, gestión de riesgos, vigilancia sanitaria, modelos, Colombia, Health risk, risk management, health surveillance, models, Colombia, Risco sanitário, gestão de riscos, vigilância sanitária, modelos, Colômbia

## Abstract

En Colombia, a partir de la Resolución 1229 de 2013 del Ministerio de Salud, se estableció que la inspección, vigilancia y control (IVC) sanitario debían realizarse con enfoque de riesgos. Así, en 2014, el Instituto Nacional de Vigilancia de Medicamentos y Alimentos (INVIMA) diseñó e implementó un modelo de vigilancia sanitaria basado en riesgos llamado IVC-SOA. Este modelo mide el riesgo de los medicamentos, dispositivos médicos, alimentos y cosméticos considerando tres factores: la severidad del producto (S), la ocurrencia de falla del producto (O) y la población potencialmente afectada (A), de allí su nombre SOA.

El modelo incorpora 40 variables y métodos estadísticos que permite crear un perfil de riesgos para cada una de las entidades que permite crear un perfil de riesgos para cada una de las entidades vigiladas y así generar un ranking para determinar cuáles de estas deben ser inspeccionadas.

El objetivo de este informe es presentar la metodología y los resultados obtenidos luego del diseño e implementación del modelo IVC-SOA creado por la agencia reguladora en Colombia y su efecto en la efectividad de la vigilancia sanitaria.

## Abstract (en)

Colombian Ministry of Health Resolution 1229 of 2013 established that health inspection, surveillance, and control (IVC, Spanish acronym) should be based on a risk-focused approach. In 2014 Colombia’s National Food and Drugs Surveillance Institute (INVIMA) designed and implemented a risk-based health surveillance model called IVC-SOA. This model measures the risks of drugs, medical devices, food, and cosmetics by taking into account three factors: severity of the product (S), occurrence of product failure (O), and the potentially affected population (A) - hence its name, SOA.

The model incorporates 40 variables and statistical methods that make it possible to create a risk profile for each entity surveyed, and thus to generate a ranking to determine which should be inspected.

The objective of this report is to describe the methodology and results obtained following the design and implementation of the IVC-SOA model created by the regulatory agency in Colombia, and its impact on health surveillance effectiveness.

## RESUMEN

A Resolução 1229 de 2013 do Ministério da Saúde da Colômbia estabeleceu que a inspeção, vigilância e controle (IVC) sanitários devem ser realizados com um enfoque de riscos. Assim, em 2014, o Instituto Nacional de Vigilância de Medicamentos e Alimentos (INVIMA) projetou e implementou um modelo de vigilância sanitária baseado em riscos, denominado IVC-SOA. Este modelo avalia o risco dos medicamentos, dispositivos médicos, alimentos e cosméticos com base em três fatores: severidade (S), ocorrência de falha do produto (P) e população potencialmente afetada (A), daí a sigla SOA.

O modelo inclui 40 variáveis e métodos estatísticos que permitem traçar um perfil de riscos para cada uma das entidades em vigilância e gerar um ranking para determinar quais devem ser inspecionadas.

O objetivo deste relatório é apresentar a metodologia e os resultados obtidos após o projeto e implementação do modelo IVC-SOA desenvolvido pelo órgão regulador da Colômbia e o impacto resultante na efetividade da vigilância sanitária.

El término *riesgo* se define como el efecto de la incertidumbre sobre los objetivos, o la combinación de la probabilidad de un suceso y de su consecuencia (1).

La gestión de riesgos es un tema que data de muchos años. En la Antigüedad, 2 500 años antes de Cristo, los mercaderes chinos distribuían sus mercancías en varias embarcaciones para evitar la quiebra ante un eventual naufragio. Lo que ellos hacían de manera intuitiva era reducir la probabilidad y el impacto en caso de siniestro.

Hoy en día, la gestión de riesgos se realiza de manera formal, a través de normas y estándares internacionales, tales como la ISO 31000; los cuales incluyen la identificación, evaluación, tratamiento, aceptación y comunicación de riesgos (1). Esta norma incorpora el estándar australiano neozelandés AS NZ 4360 (2).

En sectores como el financiero, la valoración de riesgos ha tenido mayor evolución, y hoy se cuenta con modelos matemáticos cuantitativos basados en el Acuerdo de Basilea II de 2004 (3).

En relación con los productos de consumo objeto de vigilancia sanitaria en alimentos, la Autoridad Europea de Seguridad Alimentaria (EFSA, por sus siglas en inglés) define tres componentes para el análisis de riesgos: la evaluación, la gestión y la comunicación de riesgos (4). A su vez, la Administración de Medicamentos y Alimentos de los Estados Unidos (FDA, por sus siglas en inglés) destaca, en su modelo de riesgos, la importancia de la vigilancia en las etapas de premercado y de posmercado (5).

Las autoridades sanitarias deben realizar inspección, vigilancia y control (IVC) a los establecimientos que están bajo su supervisión. Se trata de un proceso sistemático y constante de verificación de estándares de calidad, monitoreo de efectos en salud y acciones de intervención en las cadenas productivas orientadas a minimizar riesgos, daños e impactos negativos para la salud humana por el uso de consumo de bienes y servicios (6).

La inspección sanitaria tradicionalmente se enfoca en realizar visitas a la totalidad de establecimientos que estuviesen bajo su jurisdicción —enfoque por cobertura—, sin considerar ningún factor, criterio o variable basado en riesgo, que le permita optimizar su proceso de IVC.

El nuevo enfoque basado en riesgos genera varios desafíos para los gobiernos de la región, en especial para sus agencias reguladoras: por un lado, el apego al tradicional enfoque por cobertura, que ha venido aplazando la implementación de métodos basados en riesgos que proveen mayor información sobre las condiciones sanitarias de las industrias y sus productos; estos métodos facilitan la intervención oportuna de las autoridades sanitarias ante situaciones que potencialmente pongan en riesgo la salud de la población. Por otra parte están los altos costos operativos que asumen las agencias sanitarias para la inspección de todos sus vigilados, inclusive los de bajo riesgo o aquellos que tienen todas las certificaciones en buenas prácticas y en inocuidad.

El modelo IVC-SOA, diseñado por la agencia sanitaria colombiana INVIMA, mide los riesgos de los productos, considerando tres factores: la severidad (S), la ocurrencia (O) y la afectación (A). La severidad está asociada al impacto de un eventual daño derivado por las características intrínsecas del producto. La ocurrencia se determina por la frecuencia de falla que tenga el producto; aquí son relevantes las reacciones adversas a los medicamentos (RAM) y los resultados de laboratorio no conformes del producto. Por su parte, la afectación se mide según la población que eventualmente puede sufrir daños si falla el producto; esta se calcula teniendo en cuenta la cantidad de personas expuestas y si tienen alguna condición de vulnerabilidad, como es el caso de niños y mujeres embarazadas.

El modelo no solo valora el riesgo por producto, sino que incorpora 40 variables que miden el riesgo por establecimiento, a partir de información histórica sanitaria y datos externos especializados. Con esta información y mediante la aplicación de métodos estadísticos y matemáticos, el modelo construye perfiles de riesgo para cada establecimiento vigilado, el cual es útil para priorizar las visitas de inspección y para el seguimiento de los principales factores de riesgos sanitarios (7).

En los últimos dos años, y a partir de la implementación del modelo IVC-SOA, la agencia sanitaria aumentó su indicador de efectividad en el control sanitario; esto significa que aumentó el número de medidas sanitarias (destrucción y recogida de productos, suspensión y cierre de actividades, entre otros) con respecto a la cantidad de visitas de inspección realizadas. De esta manera, contribuye en forma directa a la protección de la salud de la población colombiana.

Este informe presenta la metodología y los resultados del modelo-IVC SOA diseñado e implementado por el INVIMA, en su papel de agencia sanitaria reguladora de Colombia. Como resultado de la implementación del modelo basado en riesgo, se optimiza la gestión y se logra un aumento en la efectividad de la agencia sanitaria.

## DISEÑO Y MÉTODOS DEL MODELO IVC-SOA

El modelo IVC-SOA tiene un enfoque holístico que integra diversos componentes y fuentes de información utilizadas en la vigilancia sanitaria de los productos objeto de IVC por parte del INVIMA. El modelo recoge información de visitas de inspección a establecimientos, de los programas de farmacovigilancia en medicamentos, tecnovigilancia en dispositivos médicos y riesgos químicos en alimentos. Además, incluye información sobre alertas sanitarias locales e internacionales, resultados de análisis de laboratorio, medidas y sanciones sanitarias, evaluaciones de buenas prácticas de manufactura y certificaciones homólogas^2^, así como quejas y denuncias sobre los establecimientos y productos.

El modelo IVC-SOA basado en riesgos obedece a un diseño propio del INVIMA, construido durante un año por diferentes especialistas de diversas profesiones como ingenieros, médicos, estadísticos, economistas, veterinarios, químicos y bacteriólogos, entre otros.

La implementación del modelo fue gradual: inició en junio de 2014 y terminó en junio de 2015. Se crearon perfiles de riesgo para más de 13 000 establecimientos vigilados. Más tarde, dichos perfiles de riesgo se actualizan con las actividades de inspección y vigilancia que se desarrollen durante cada trimestre.

El Modelo IVC-SOA está estructurado de la siguiente manera: siete variables transversales o comunes para todos los tipos de productos, 33 variables propias o específicas por producto y 40 riesgos que son aplicados según el tipo de producto. También se califican los productos según su severidad, ocurrencia y afectación (SOA). Todos los puntajes de riesgos mencionados se totalizan en el índice de riesgo agregado (IRA), que establece el valor de riesgos de cada uno de los establecimientos vigilados. Las variables transversales y las propias evalúan el riesgo por establecimiento o entidad, mientras que el SOA mide el riesgo por producto. Queda así conformado un modelo de riesgo mixto que evalúa tanto los establecimientos como los productos.

El valor de riesgo para cada establecimiento vigilado, está dado por la siguiente ecuación:

*IRA_i_ = β_1_.VT_i_ + β_2_.VP_i_ + β_3_.Riesgo SOA_i_*

Donde:

IRA_i_ es el nivel de riesgo acumulado de un establecimiento_i_, derivado de las variables transversales, las variables propias y el riesgo SOA por producto. Este valor servirá de referencia para determinar la urgencia con la cual un establecimiento deba recibir inspección sanitaria.

VT_i_ es el puntaje de riesgo acumulado de un establecimiento_i_, derivado de las variables transversales o comunes de todas las direcciones misionales del Instituto (áreas funcionales): alimentos, medicamentos, dispositivos médicos y cosméticos. Por ejemplo: cumplimiento de estándares sanitarios, medidas sanitarias y fecha de la última visita, entre otras.

VP_i_ es el puntaje de riesgo acumulado de un establecimiento_i_, derivado de las variables propias de cada dirección (particulares por tipo de producto). Por ejemplo: enfermedades transmitidas por alimentos (ETA, alimentos), infecciones transmitidas por transfusiones (ITT, bancos de sangre), reacciones adversas por medicamentos (RAM, medicamentos), entre otras.

Riesgo SOA se refiere al puntaje obtenido después de calificar los riesgos de cada uno de los productos de un establecimiento_i_, según su severidad, ocurrencia de falla (ocurrencia) y afectación (población expuesta).

β_1_, β_2_ y β_3_ son los ponderados o pesos de cada componente, su suma debe ser igual a uno. Estos se calculan a través del método estadístico de análisis de componentes principales (ACP^3^) para datos categóricos (8, 9).

Las variables transversales utilizadas son siete: tipo de actividad de la cadena productiva, cumplimiento de estándares sanitarios, tiempo transcurrido de la última visita, medidas sanitarias, histórico de denuncias, registros sanitarios y responsable técnico ante la autoridad sanitaria (este último es el profesional que ampara o asiste técnicamente a un establecimiento vigilado en su proceso productivo). En cuanto a las variables propias, solo se citan algunas como ejemplo: certificación HACPP (siglas en inglés correspondiente a Análisis de Peligros y Puntos Críticos de Control) en alimentos, tenencia del programa de farmacovigilancia en medicamentos, resultado de la señalización en dispositivos médicos, selección de donantes en bancos de sangre, entre otras. Las demás variables propias del modelo se citan en el Anexo .

Se realizó un análisis multivariado del modelo, analizando estadísticamente la frecuencia, varianza y correlación de sus variables (10–15). Además, se crearon “variables componentes” para totalizar las variables transversales, propias y el SOA por producto. Se utilizó el método de análisis de componentes principales para datos categóricos para calcular los ponderados de las variables ordinales, y el análisis factorial para los ponderados de las “variables componentes” que son continuas (8, 9, 14).

Los valores de las variables tienen una métrica estandarizada, sus puntajes están definidos entre 1 y 5 (5 es el máximo valor en riesgos). Estos valores se asignan según los datos estadísticos, el historial del establecimiento vigilado y el tipo de productos que fabriquen o importen. Para la definición de las variables se creó un Comité de Riesgos, donde participaron especialistas de las áreas técnicas de la agencia sanitaria.

En el cuadro 1 se explica cómo se califica una industria de derivados lácteos con el modelo IVC-SOA. El puntaje asignado a cada variable depende de la situación observada en cada establecimiento al momento de realizar la inspección sanitaria, también influyen su historial sanitario y la información allegada por los usuarios, consumidores o autoridades sanitarias. Los establecimientos que tengan mayor exposición sanitaria, tendrán mayor calificación del riesgo.

**CUADRO 1.** Calificación de riesgo de un establecimiento con el modelo IVC-SOA

Tipo de variables	Peso de la variable (%)	Calificación de riesgo (de 1 a 5)	Justificación de la calificación según tablas de referencia de riesgos
	Variables transversales (VT) (β1 = 0,45)		
VT1. Tipo de actividad de la cadena productiva	11,24	4	Fabrica, almacena y distribuye (nivel de complejidad)
VT2. Cumplimiento de estándares sanitarios	18,52	3	Favorable con observaciones (valor intermedio)
VT3. Tiempo transcurrido desde la última visita	5,08	3	Dos años y dos meses (más de cuatro años corresponde a un puntaje de 5 en riesgos)
VT4. Medidas sanitarias aplicadas al establecimiento	22,50	2	Una medida en los últimos 3 años
VT5. Histórico de denuncias asociadas al establecimiento	13,43	3	Una denuncia por publicidad
VT6. Número de registros por establecimientos	14,32	2	1 para quesos frescos, 1 para quesos madurados
VT7. Responsable técnico ante la autoridad sanitaria	14,90	5	No tiene responsable técnico, el cargo está vacante
**Riesgo de las variables transversales**		**3,04**	
	Variables propias de alimentos VP (β2 = 0,25)		
VP1. Tipo de establecimiento	37,00	5	Derivados lácteos (lácteos de alto riesgo)
VP2. Reporte ETA	53,00	4	1 ETA en 1 año
VP3. Certificación HACPP INVIMA	10,00	3	No certificada HACCP
**Riesgo de las variables propias**		**4,27**	
	Riesgo SOA por producto (β3 = 0,30)		
R1: Riesgo de alteración de la calidad del producto por falla microbiológica			
Severidad (S)		5	Lácteos = 5; clasificación del producto según su riesgo
Ocurrencia (O)		3	Dos rechazos de laboratorio por *Listeria monocytogenes*
Afectación (A)		5	Población vulnerable (hogar de niños)
**S x O x A**		75	Factor SOA
**Puntaje SOA estandarizado (de 1 a 5)**		**4,22**	SOA con valores de 1 a 5
**Índice de riesgo agregado (IRA)** **^a^**		**3,70**	Riesgo alto

a IRA = β1.VT + β2.VP + β3. Riesgo SOA, se obtiene con el promedio ponderado entre los βetas y los puntajes de riesgos de VT, VP y Riesgo SOA. (0,45 X 3,04 + 0,25 X 4,27 + 0,30 X 4,22).

β1, β2 y β3, corresponde a los ponderados de las variables transversales, variables propias y riesgo SOA, respectivamente. La suma debe ser igual a 1. Estos valores se calculan mediante el método de análisis de componentes principales (CPA).

ETA, enfermedades transmitidas por alimentos; HACPP, siglas en inglés correspondientes a Análisis de Peligros y Puntos Críticos de Control; INVIMA, Instituto Nacional de Vigilancia de Medicamentos y Alimentos.

***Fuente:*** elaboración propia de la Unidad de Riesgos (Dirección General, INVIMA, Colombia).

Por ejemplo, en la variable transversal “VT1: tipo de actividad de la cadena productiva” el puntaje de riesgo dependerá del nivel de complejidad de la industria: cuantas más actividades desarrolle, mayor será su nivel de riesgo. En esta variable, las actividades de la cadena productiva, son: investigación y desarrollo, fabricación, envase, empaque secundario y acondicionamiento, dispensación, importación, almacenamiento, distribución y comercialización. En el caso citado, el establecimiento de lácteos fabrica, almacena y distribuye; según las tablas de referencia, el puntaje que le corresponde es de 4, con 5 como el mayor valor. De forma análoga se analizan las demás variables: cuanto mayor sea la frecuencia de falla o exposición, mayor será su calificación.

En relación con las variables propias, se citan en el ejemplo: “VP1: tipo de establecimiento según producto”, “VP2: reporte de ETA” y “VP3: certificación HACPP”. Estas variables solo aplican a la industria alimenticia, por eso su característica de “propias”; cada tipo de producto tiene sus variables particulares. En el cuadro se muestra que el establecimiento de estudio produce lácteos y según la tabla de referencia su valor es de 5, es decir de alto riesgo (VP1). También tiene reportada una ETA en el último año, por eso su calificación de 4 (VP2); y por último no tiene certificación HACCP, la cual es un proceso voluntario y por eso tiene calificación de 3 (VP3).

Para la calificación del componente “Riesgo SOA” se consideran los riesgos que tienen los tipos de producto que fabrica un establecimiento. Cada riesgo es calificado por su severidad, ocurrencia y afectación. En el ejemplo citado, de los 40 riesgos que contiene el modelo, se seleccionó “alteración de la calidad del producto por falla microbiológica”. En este caso, la severidad se calificó con 5 porque, según la Resolución 719 de 2015 del Ministerio de Salud de Colombia, los lácteos están clasificados como alimentos de alto riesgo. La ocurrencia tiene calificación 3 porque, según la tabla de referencia, ese es el valor que le corresponde a dos rechazos de laboratorio en los últimos tres años; y por último, la afectación tiene calificación de 5 porque ese producto de ese establecimiento, es consumido por una población vulnerable de un hogar de niños de escasos recursos.

Las calificaciones de severidad, ocurrencia y afectación se multiplican y a ese resultado se le aplica raíz cúbica para estandarizar el resultado en valores de uno a cinco.

Cabe anotar que cada componente (variables transversales, variables propias y riesgo SOA) tiene una ponderación que depende de la variabilidad de los datos. Según el ejemplo: 0,45 para VT; 0,25 para VP y 0,30 para riesgo SOA. Estos valores son calculados con el método estadístico de análisis de componentes principales para datos categóricos. Este mismo método se aplica a las variables que conforman cada componente (variables transversales y propias).

En el cuadro 2, se observa que el índice de riesgo agregado (IRA) se obtiene con la suma de los productos ponderados de las variables transversales, variables propias y riesgo SOA. En este caso, el puntaje obtenido es de 3,70 sobre 5; que equivale a riesgo alto. La franja de valores entre 4 y 5, corresponde a riesgo muy alto; entre 3 y 4 puntos corresponde a riesgo alto, entre 2 y 3 puntos corresponde a riesgo moderado; y entre 1 y 2 a riesgo bajo. Hoy en día, el INVIMA ha calculado para todos los establecimientos objeto de vigilancia sanitaria, un índice de riesgo agregado (IRA) para la priorización de sus actividades de inspección, vigilancia y control.

**CUADRO 2.** Cálculo del Índice de riesgo agregado (IRA) con el Modelo IVC-SOA

Componente	Variables de riesgos	B_i_
Variables transversales (Vti)	3,04	0,45
Variables propias (Vpi)	4,27	0,25
Riesgo SOAi	4,22	0,30
**IRAi** **^a^**	**3,70**	1,00

a Calculado según la fórmula: β1.VTi + β2.VPi + β3 Riesgo SOAi

βi, ponderados de las variables transversales, variables propias y riesgo SOA respectivamente; i, establecimiento.

***Fuente:*** elaboración propia de la Unidad de Riesgos (Dirección General, INVIMA, Colombia).

Además, el modelo calcula el índice de riesgo agregado corporativo (IRAC). Este índice consolida los puntajes IRA de los diferentes establecimientos según el tipo de producto (alimentos, medicamentos, dispositivos médicos y cosméticos). Este índice ofrece, en una sola cifra, el nivel de riesgo de todos los establecimientos vigilados. A partir de este índice, se establecen estrategias de vigilancia y mejoramiento sanitario para los establecimientos vigilados. Este valor se calcula aplicando el percentil 90 del IRA de todos los establecimientos.

## RESULTADOS

Implementado el modelo en el año 2014, se estableció un indicador de efectividad para medir el porcentaje de medidas sanitarias aplicadas en relación con el número de establecimientos visitados por año, para conocer los efectos en la inspección y control sanitario.

**FIGURA 1.**
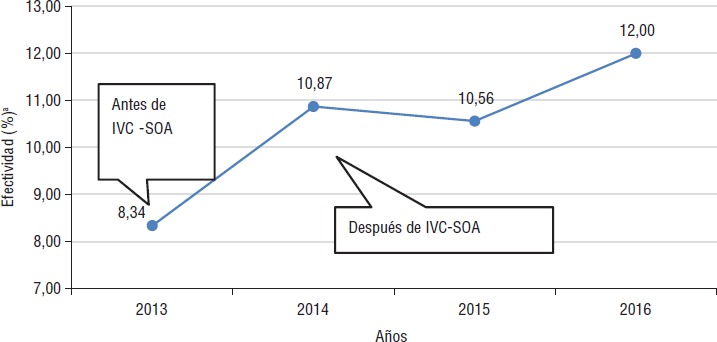
Indicador de efectividad del modelo IVC-SOA

^a^ El porcentaje de efectividad se calcula dividiendo el número de medidas sanitarias por el número de visitas de inspección multiplicado por 100.

En la figura 1 se muestra cómo aumentó el porcentaje de efectividad en el control sanitario después de la implementación del modelo IVC-SOA en 2014. Esto significa que el porcentaje relativo de medidas sanitarias pasó de un 8,34% del año 2013 (sin el modelo) a más del 10% y hasta el 12% en el año 2016 con la implementación del modelo; lo que aumentó el grado de asertividad al momento de escoger un establecimiento vigilado para la inspección.

***Fuente:*** elaboración propia de la Unidad de Riesgos, Dirección General, INVIMA, Colombia.

Como resultados de su implementación, el modelo IVC-SOA del INVIMA:

- Estandarizó y homologó el concepto sobre la gestión de riesgos al interior de la entidad.
- Mejoró el proceso de inspección, vigilancia y control sanitario, haciéndolo más eficaz y eficiente.
- Estableció un índice de riesgo sanitario llamado índice de riesgo agregado (IRA), el cual sirve de referencia (línea base) para la evaluación de las estrategias sanitarias de la entidad.
- Permitió establecer perfiles de riesgo para cada uno de los establecimientos y tipos de productos que están bajo vigilancia sanitaria. A partir de estos resultados, se priorizan las visitas de inspección para los establecimientos de mayor riesgo y se aplica el método de “autoevaluación sanitaria”^4^ para los de bajo riesgo.
- Permitió realizar el seguimiento y monitoreo de las principales variables y riesgos sanitarios (vigilancia).
- Generó información sanitaria de los establecimientos por regiones o zonas geográficas (georreferenciación) útil
- Facilitó la toma de decisiones en cuanto a políticas, estrategias e intervención sanitaria.

## DISCUSIÓN

Al corte de junio 30 de 2016, el INVIMA tenía a cargo la vigilancia de 13 413 industrias que fabrican, importan o administran más de 1 800 diferentes tipos de productos. De ese total, 59% corresponde a industrias de alimentos, 19% a fabricantes e importadores de dispositivos médicos, 8% a la industria cosmética, 7% a la industria farmacéutica y 6% a plantas de sacrificio animal de bovinos, porcinos, aves y otras especies (frigoríficos). También vigila 83 bancos de sangre y 18 bancos de tejidos encargados de proveer tejido cardiovascular, tejido corneano y ocular, membrana amniótica, tejido osteomuscular y piel.

En el cuadro 3 se observa que, de un total de 13 413 establecimientos vigilados, 40,54% se encuentran en riesgo alto, 51,27% en riesgo moderado y 8,1% en riesgo bajo. También se aprecia que dos empresas de alimentos, un frigorífico, un establecimiento de dispositivos médicos, cinco bancos de tejidos y tres bancos de sangre están en riesgo muy alto, lo que requiere vigilancia especial. Asimismo, se observa que de 7 922 fábricas de alimentos, 4 522 se encuentran en riesgo alto (57,1%). A su turno, en la industria de cosméticos, de 1 103 establecimientos, 897 tienen riesgo moderado, es decir el 81,3%. Esta clasificación se realiza con base en el índice de riesgo agregado (IRA). Adicional a esta información, se generan mapas por región geográfica según el nivel de riesgo de los establecimientos (16).

**CUADRO 3.** Número de establecimientos según el nivel de riesgo sanitario, con corte al 30 de junio de 2016

Tipos de fábrica o establecimiento	Número de establecimientos y porcentajes según el nivel de riesgo					Porcentaje por tipo de fábrica (%)
	Nivel de riesgo				Total	
	Muy alto	Alto	Moderado	Bajo		
Alimentos	2	4 522	3 393	5	7 922	59,0
Frigoríficos	1	105	670	2	778	6,0
Cosméticos	0	143	897	63	1 103	8,0
Medicamentos	0	119	658	132	909	7,0
Dispositivos médicos	1	516	1 198	885	2 600	19,0
Bancos de tejidos	5	6	7	0	18	0,1
Bancos de sangre	3	26	54	0	83	0,6
Total	12	5 437	6 877	1 087	13 413	100
Porcentaje por nivel de riesgo (%)	0,09	40,54	51,27	8,1	100	

Por otra parte, en junio de 2016, el IRAC terminó con un valor de 3,41; esto significa que 90% de los establecimientos vigilados tienen un valor de riesgos menor o igual a 3,41 (con 5 como el mayor valor en riesgo); el restante 10% de establecimientos tiene puntajes superiores, lo que exige un mayor seguimiento y vigilancia.

### Conclusión

En el año 2014, el INVIMA diseñó e implementó un modelo de inspección, vigilancia y control con enfoque de riesgos, llamado IVC-SOA. Este modelo evalúa los productos según su severidad, ocurrencia de falla y población potencial afectada; también califica los establecimientos utilizando 40 variables construidas a partir de fuentes de información interna, externa y datos históricos sanitarios. Este modelo utiliza ecuaciones matemáticas y métodos estadísticos para la valoración del riesgo sanitario.

El modelo IVC-SOA establece un *ranking* con los establecimientos de mayor riesgo, el cual es fundamental para la programación de visitas de inspección y la vigilancia sanitaria.

El modelo IVC-SOA aumentó el porcentaje de efectividad en el control sanitario, lo que se traduce en una mayor cantidad de medidas sanitarias debido a una mejor selección de los establecimientos a inspeccionar con base en sus perfiles de riesgo y mejora en la gestión institucional de la agencia reguladora.

El modelo IVC-SOA es un aporte a los esquemas de vigilancia sanitaria de la región; al integrar calificaciones de riesgos por productos e industria en una sola ecuación. Asimismo, su operación se fundamenta en conceptos de matemáticas y estadística aplicados para la valoración del riesgo sanitario. Además su enfoque holístico le permite a la Agencia sanitaria evaluar los riesgos de manera sistémica

### Agradecimientos.

Los autores agradecen a los doctores Francisco Javier Sierra y Alexandra Esteban del INVIMA, por sus aportes y comentarios al documento.

### Declaración.

Las opiniones expresadas en este manuscrito son responsabilidad de los autores y no reflejan necesariamente los criterios ni la política de la RPSP/PAJPH y/o de la OPS.

## Footnotes

2 Se evalúa el cumplimiento de los estándares sanitarios según el tipo de producto: Buenas Prácticas de Manufactura, BPM (alimentos, medicamentos), Buenas Prácticas de Laboratorio, BPL (laboratorios), Buenas Prácticas de Elaboración, BPE (hospitales), Buenas Prácticas de Almacenamiento, BPA (importadores de dispositivos médicos) y Certificado de Capacidad de Producción, CCP (cosméticos).

3 El método de las componentes principales (ACP) es un procedimiento estadístico que permite construir un conjunto de variables no correlacionadas a partir de la combinación de variables correlacionadas. Las nuevas variables conocidas como componentes principales son combinaciones lineales de las variables originales. A partir de este método es posible determinar el porcentaje de variabilidad total que explica cada una de los componentes principales. Las variables de mayor variabilidad tendrán un mayor peso o porcentaje en relación con las demás.

4 Autoevaluación sanitaria es una estrategia de vigilancia que se aplica a los establecimientos de bajo riesgo; estos deben diligenciar una Guía Sanitaria y enviarla a la Agencia para su verificación. para el análisis de causalidad de los factores de riesgo sanitario.
